# Emotional Reactivity and Family-Related Factors Associated With Self-Injurious Behavior in Adolescents Presenting to a Child and Adolescent Psychiatric Emergency Service

**DOI:** 10.3389/fpsyt.2021.634346

**Published:** 2021-06-09

**Authors:** Stephanie Kandsperger, Irina Jarvers, Angelika Ecker, Daniel Schleicher, Joseph Madurkay, Alexandra Otto, Romuald Brunner

**Affiliations:** Clinic of Child and Adolescent Psychiatry, Psychosomatics and Psychotherapy, University of Regensburg, Regensburg, Germany

**Keywords:** emotional reactivity, suicidal behavior, self-injurious behavior, non-suicidal self-injury, emergency, adolescents, family related factors

## Abstract

**Background:** Adolescents presenting in a child and adolescent psychiatric emergency service show various psychiatric disturbances, most commonly suicidal ideation, suicide attempts, and non-suicidal self-injury (NSSI). It was postulated that especially disturbed emotion regulation contributes to self-injurious behavior of young people. This study aims to investigate the relevance of emotional reactivity (ER), as part of emotion regulation, during an acute crisis, how it relates to self-injurious behavior reinforcement and how a family as well as peers' history of self-injurious behavior are associated with self-injurious behavior of presenting adolescents. Additionally, crisis-triggering background factors were evaluated from the perspective of patients and their caregivers.

**Methods:** A consecutive sample of 86 adolescents aged 11–18 years presenting to the emergency outpatient department due to self-injurious thoughts and behavior received a pretreatment psychiatric evaluation. Among other psychometric measures and structured clinical interviews, ER was measured via the Emotion Reactivity Scale (ERS). Family-related aspects were collected both through evaluation of history and through questionnaires filled in by custodians or parents.

**Results:** Data analysis revealed that suicidal ideation was significantly related to family history with self-injurious behavior in comparison with a family background without such a history. A significant positive correlation was apparent between the ERS sensitivity score and occurrence of NSSI within the past year. A relationship between the ERS and distinct types of reinforcement as a motivation factor for NSSI was found. *Post-hoc* tests revealed a significant difference between boys and girls when no positive peers' history is present with boys having lower ERS scores than girls, but no difference when both groups had friends engaging in self-injurious behavior. There was only moderate agreement between parents and their children in naming reasons for the current crisis involving NSSI.

**Conclusion:** Emotional regulation, especially ER, has an influence on patients' acute psychiatric symptomatology and when experiencing an acute crisis should be brought into focus early at psychiatric assessment. A history of self-injurious behavior taken from patient's family members and close circle of friends and agreement on reasons for the crisis should be routinely included in the exploration of a patient presenting with self-injurious behavior.

## Introduction

A psychiatric emergency is defined as an acute disturbance of a patient's mood, thought or behavior, by which the individual may cause harm to either himself or others (1). Initial support for those patients, in many cases, is psychiatric emergency care (2, 3), where circumstances for urgent presentations by young people range from small events to life-threatening situations (4, 5). Multiple reasons are presented for emergency appearances, the main symptoms are self-injurious thoughts and self-injurious behaviors (6). Self-injurious thoughts and behaviors (SITB) range from non-suicidal self-injury (NSSI), which is carried out with no intention to die, to suicidal behavior, in which the individual has at least some intention to die (7). Although NSSI and suicidal behavior must be distinguished in terms of motivation/intention and medical severity, there is a high and not negligible overlap between the two behaviors (8), which supports a combined examination in an emergency setting.

While the prevalence of SITB up to puberty is low (9), these symptoms develop into a very common pattern of behavior during adolescence. In a large sample of adolescents from European countries, an overall lifetime prevalence of direct self-injurious behavior (regardless of suicidal intent) of about 27.6% could be found (8). In child and adolescent psychiatric hospitals, the prevalence rate of NSSI is as high as 60% of admitted patients (10). NSSI is understood as self-inflicted, voluntary, direct injury, or damage to body tissue without conscious suicidal intention, which is not socially accepted (11). Although NSSI can manifest itself without mental illness, it is often associated with emotional abnormalities and behavioral problems (12). Those affected show a high degree of suffering and in addition NSSI is linked to affective (8) and personality disorders, particularly borderline personality disorder (BPD) (13). Self-injurious behavior among young people applies as an important risk factor for the prediction of a borderline personality disorder (14). Unfortunately, many parents have no knowledge of NSSI and its treatment and thus suffer from great emotional stress because of it (15). An additional risk factor is the strong association between NSSI and suicidal behavior (16). In some cases, NSSI is a potent predictor of suicide attempts, which underlines the importance in preventing these behaviors (17–19).

Teenagers who reported frequent suicidal ideation showed an 18-fold increased risk of NSSI (20). It was revealed that stopping self-injurious behavior reduces the risk of suicidal ideation and behavior in adolescents and therefore contributes to reducing further suicidal behavior (16), which can be defined as suicidal ideation, suicide plans, suicide attempts, and completed suicides (21). Suicidal behavior tends to be recurrent and can be a harbinger of the completion of suicide (22). The transition between different self-injurious thoughts and behaviors is quite fast and an important time-frame for prevention and intervention is between 6 and 12 months after the occurrence of suicidal ideation (23). Thus, emergency care is crucial for the prevention and therapy of recurrent suicidal ideation. In addition, NSSI and suicidal behavior have been inserted into section Results of the new DSM-5 (24, 25), which supports the relevance of a combined approach.

Research on the origin of suicidal behavior suggests for it to be familial and liability for suicidal behavior in families is apparently transferred independently of the psychiatric disorder itself (26, 27). In addition, a suicide attempt and suicide completion of friends is considered a risk factor for one's own suicidal behavior (28). A recently attempted suicide of a friend is a significant predictor for a future suicide attempt (29). Furthermore, exposure to self-injurious behavior of family members and friends is associated with adolescents' own self-injurious behavior (30, 31). NSSI of teenagers has been reported to be associated with an increasing perception of their friends' involvement in depressive/self-injuring thoughts and behaviors (32). This result has also been replicated in young adults (33).

Apart from NSSI and suicidal behavior within the familial and social network, also other aspects have been identified as determinants for NSSI during youths' development. Numerous studies have demonstrated the importance of emotional dysregulation for development and the clinical course of mental disorders (34). The results of In-Albon et al. (35) illustrate that adolescents with NSSI compared to a healthy control group have difficulties with their emotion regulation in certain areas. In addition, compared to a similarly psychiatrically affected clinical control group, adolescents with NSSI had significantly more difficulties in the regulation of their emotions, particularly impulse control, clarity about one's own feelings, goal-oriented behavior and difficulties in accessing emotion regulation strategies (35). It is therefore surprising that the assessment of emotion regulation strategies has so far been poorly integrated into the diagnosis of mental disorders (34). A study by Glenn and Klonsky (36) also showed that the NSSI disorder explains a unique proportion of variance in almost all aspects of emotion dysregulation (36).

Emotional reactivity (ER) is part of the emotional-response-process; an individual considers a situation, rates it as relevant and feels the activation of an emotion that can be described in terms of experience, behavior and physiology (37). Nock et al. (38) specified ER as “the extent to which an individual experiences emotions (a) in response to a wide array of stimuli (i.e., emotion sensitivity), (b) strongly or intensely (i.e., emotion intensity), and (c) for a prolonged period of time before returning to baseline level of arousal (i.e., emotion persistence).” According to this concept increased ER results in emotion regulation difficulties (38). In line with these results, individuals who engage in NSSI report higher ER compared to a healthy control group (39). To the best of our knowledge, previous studies in clinical (40) and non-clinical samples (39, 40) have not examined the importance of ER in the aftermath of an acute crisis. Thus, conclusions cannot be drawn as to whether increased ER is a key factor in maladaptive emotion regulation after crisis situations.

Patients with NSSI often engage in this behavior when confronted with overwhelming negative emotions (41). In accordance with the model outlined by Nock (7), apart from automatic negative reinforcement (intrapersonal), i.e., NSSI is followed by an immediate reduction or a cessation of aversive feelings, there are three other important functions by which NSSI can be perpetuated (42). The second intrapersonal aspect is automatic positive reinforcement (i.e., behavior is followed by occurrence or increase in desired feelings) (7, 42). NSSI can also be maintained by interpersonal factors, namely social positive reinforcement (i.e., occurrence or increase of a desired social event), but also by social negative reinforcement (i.e., decrease or termination of a social event) (7, 42). In a group of child psychiatric inpatients aged 12–19 years, most adolescents reported that they engaged in NSSI to get away from bad feelings (43). NSSI is often reported as a coping strategy to handle negative emotional states better (44). Thus, the current research question is specifically about NSSI and whether higher ER is associated with the assumed four significant functions just described above.

Not only ER plays a crucial role, but also the social environment. In a school-based survey, friends were significantly more likely than family to be asked for support when engaged in NSSI (45). In general, seeking official services or medical professionals was even more difficult for youths and many young people felt that they should be able to cope on their own and were concerned that seeking help would cause them more problems and hurt people they cared about (45). For this reason, we were interested in whether caregivers' and patients' explanatory models agree or not. We assumed that if there is a high degree of agreement, adolescents are more likely to open up to the caregivers (46) and, in the best case, to be motivated by the caregivers to allow expert support. However, our daily routine work with adolescent patients leads us to believe that the reasons given differ between caregivers and patients which leads to a significant impediment to a successful therapeutic intervention.

Adolescents who are admitted to an emergency service at clinics of child and adolescent psychiatry show a broad range of psychopathological disorders, but above all primarily suicidal behavior and NSSI. The present study aims at investigating the extent of young people's psychiatric burden and the extent to which ER is conspicuous even in the case of an acute crisis. Given the state of current knowledge, we proposed the following hypothesis that youths with NSSI have increased ER measured via the Emotion Reactivity Scale (ERS). Furthermore, we expected a significant relationship between ERS scores and both positive and negative automatic reinforcement, as it is most commonly reported as a motivation for NSSI. In addition, we also focused on psychiatric family history with regards to psychological/psychiatric illnesses (including suicide, attempted suicide, NSSI) and the occurrence of suicide attempts or NSSI among close friends (peers' history). Here we assumed an association between a positive family or peers' history on the occurrence of thoughts and actions of NSSI as well as suicidal ideation and suicide attempts. Lastly, it seems important for the management of crisis situations to know which explanatory models for the emergence of the acute crisis the adolescents themselves and their caregivers claim and whether these models agree or contradict each other. It was hypothesized that the reasons for the crisis given by guardians and patients differ significantly.

## Materials and Methods

### Participants and Recruitment

Eighty-six patients [75.6% female, age M = 15;8 (years; months)] were recruited via the emergency outpatient department of the Clinic of Child and Adolescent Psychiatry, Psychosomatics and Psychotherapy of the University Regensburg, Germany. This clinic is a typical child and adolescent psychiatric hospital of maximum care. Patients between 11 and 19 years, who are presented for emergencies during the day and at night because of SITB, are offered a standardized emergency management with specified diagnostic assessments and short-term intervention by means of two further time points (time point 2 and time point 3). The existing standardized emergency management is characterized by rapid time point allocation [time interval between emergency appointment and time point 2: M = 6.59 days (SD = 4.67 days), time interval between emergency appointment and time point 3: M = 17.12 days (SD = 9.43 days)] and including an initial early intervention for adolescents with NSSI and suicidal behavior with the aim of preventing an aggravation of SITB or bringing it to remission. Patients with acute psychotic disorder or other acute psychiatric conditions that could affect the patient's ability to consent, intellectual impairment (IQ lower than 80) according to clinical assessment or acute suicidal tendencies requiring prolonged inpatient treatment (more than 12 nights) on one of the hospital units were not included. The specified diagnostic assessment and short-term intervention by means of two further time points were also not offered to those patients who already receive regular outpatient treatment from established child and adolescent psychiatric services and who did not require a specified treatment offer. Thus, our sample represents a typical child and adolescent psychiatry outpatient clinic, as patients with a longer acute need for inpatient treatment were not included.

Outliers were defined as more than 3 standard deviations from the mean and were removed a priori for lifetime prevalence (*n* = 2 for suicidal ideation, *n* = 1 for suicidal attempts, *n* = 3 for NSSI thoughts, *n* = 5 for NSSI behavior) and the past year's prevalence (*n* = 2 for suicidal ideation, *n* = 1 for suicidal attempts, *n* = 1 for NSSI thoughts, *n* = 4 for NSSI behavior) for the dependent variables.

The specified standardized psychiatric assessment included a problem hierarchy to enable further recommendations to be made. In addition, a detailed safety plan was developed with the patient for prevention or support in future crisis situations. At time point 3, caregivers and patients were asked whether they agree to participate in two follow-up examinations (4 and 8 weeks after the third time point to evaluate the effectiveness of the standardized procedure). The first emergency presentation time point as well as the standardized specified assessments (time point 2) and the short-term intervention and notification of further recommendations (time point 3) were clinical procedures. The two follow-up time points represent the longitudinal part of the study design and will be reported after completion of the follow-up investigations.

The present study was approved (No.: 19-1426-101) by the ethics committee of the University of Regensburg. Participants and their caregivers gave their informed and written consent to take part in the study. If a crisis-like worsening of symptoms became apparent in one of the clinical time points or in one of the two follow-up examinations, an inpatient crisis admission was provided as an option, if necessary. The purpose of this paper is to describe the baseline sample that participated in the specified psychiatric assessments (time point 2) as well as in the short-term intervention (time point 3) and address the relationship between ER and NSSI and suicidal behaviors.

### Measures

The sociodemographic information as well as the clinical characterization with regard to NSSI and suicidal behavior, a possible concomitant borderline personality disorder and psychiatric comorbidities were performed in all patients by means of a semi-structured clinical interview with the following variables: Age/date of birth, type of school, treatment setting, relationship status of parents, type of residence, psychiatric family history. Teenagers were also asked about any close friends who showed NSSI or attempted suicide (positive peers' history). The time points 2 and 3 were handled by 4 experienced clinicians in the field of child and adolescent psychiatry. These clinicians were instructed and trained in conducting the structured clinical interviews.

Several clinical interviews were conducted on categorical and dimensional psychiatric dimensions. The psychiatric diagnoses were determined as follows: The German Version of the Mini-International Neuropsychiatric Interview for Children and Adolescents (M.I.N.I. KID 6.0), which is a short structured interview for diagnosing according to DSM-IV and ICD-10 (47), was administered by experienced clinicians. The final diagnoses were made on the basis of those results of the M.I.N.I. KID and an interactive discussion between at least two clinical experts including at least one child psychiatrists based on the clinical interviews. Also the German version of the Structured Clinical Interview for DSM-IV, Axis II (SCID-II), subsection borderline personality disorder (BPD), was conducted with patients to assess the diagnostic criteria of BPD (48). This subsection contains 9 questions according to the DSM-IV diagnostic criteria, and fulfilled criteria on at least 5 items confirm the diagnosis of BPD. The Self-Injurious Thoughts and Behaviors Interview (SITBI) (49) is a structured interview that is divided into 6 modules (including suicidal ideation, suicide plans, suicide gestures, suicide attempts, thoughts of NSSI, and NSSI itself) measuring the presence, frequency and characteristics of six types of self-injurious thoughts or behaviors. The SITBI is a well-suited diagnostic tool for clinic and research with good psychometric properties, which has also been reported for the German version of the SITBI (43). The four functions of NSSI measured in the SITBI reflect the general characteristics of reinforcement as described by Nock and Prinstein (42): Automatic negative reinforcement is queried by “getting rid of bad feelings,” automatic positive reinforcement is queried by “to feel something,” social positive reinforcement is queried by “to get attention” and social negative reinforcement is queried by “to get out of doing something”(42). Two self-questionnaires were included in the study: The German version of the Symptom Checklist-90 Revised (SCL-90-R) (50, 51) is a multidimensional self-report symptom inventory that comprises 90 items scored on a 5-point-Likert-scale from 0 to 4, which can be averaged over 9 subscales and the Global Severity Index (GSI), an indicator for general mental distress. The internal consistency, especially for the GSI is very good (α = 0.97), also for the German version (α = 0.94, −0.98) and sufficient evidence of validity has been shown (50). Additionally, the ERS was used to measure emotional sensitivity, arousal/intensity, persistence and a total ER score (38). The ERS contains 21 items (10 items for sensitivity, 7 items for arousal/intensity, 4 items for persistence) for self-report on a 5-point-Likert-scale, with preliminary evidence regarding reliability and validity by the original authors (38). Further evidence of psychometric properties was presented within a community screening assessment, highlighting the mediating, and/or reinforcing effect of ER for SITB in adults (52).

We also administered several self-developed questionnaires to the accompanying guardians. If the patients did not live at home with their parents, the questionnaires were filled in by the caregiver who could provide the most detailed information about the respective patient [e.g., legal guardian, (foster) parent or caregiver of the residential group]. We also interviewed the custodians of our patients about suffering from neurological diseases or mental illness and specifically whether attempted suicide/completed suicide and NSSI in family members had occurred. If one of these descriptors was affirmed, we defined this as positive family history. Additionally, we specifically added the pertinent parts from the SITBI (49) that query possible causes for the current crisis to the accompanying guardians in order to explore their point of view.

Furthermore, the general functional level as well as the severity of the mental illness were assessed. The psychological, professional, and social capacities were evaluated using the Global Assessment of Functioning Scale (GAF) (53). The GAF scale is divided into 10 levels of function with 10 points each. It ranges from 1 (lowest performance level) to 100 (highest performance level). The Clinical Global Impressions Scale (CGI-S) was used to assess clinical severity (54). The severity of the patient's disease was evaluated with a 7-point-Likert scale, ranging from 1 (normal) to 7 (among the most extremely ill).

### Statistical Analyses

First, as the outcome variables (suicidal ideation, suicidal attempts, NSSI thoughts, NSSI behavior) were not normally distributed and the sample size reduced due to group splits, the effect of grouping variables (sex, presence of positive family history, presence of positive peers' history) was examined through Mann-Whitney-*U*-tests. Mann-Whitney-*U*-tests have the advantage of being robust against unequal sample sizes and do not require the assumption that the dependent variable is approximately normally distributed (55). As ERS scores were normally distributed, the relationship between positive family or positive peers' history and ER was determined through an analysis of variance (ANOVA). In the subsequent procedure, the relationship between patients' ERS scores, age, and outcome variables was analyzed through bivariate correlations (Kendall's τ). Subscales of the ERS that correlated significantly while controlling for age were added as predictors into a linear regression model in order to determine the proportion of variance that could be explained by ER. In order to examine the relationship between ER and NSSI further, bivariate correlations (Kendall's τ) were computed between the ERS scores and the four items measuring the types of reinforcement that may serve as motivators for NSSI behavior. Finally, Cohen's κ was run to compare patients' reasons NSSI behavior to their guardian's reasons for the emergency presentation. According to Dunn (56) also κ_max_ was reported to demonstrate the extent to which raters' ability to agree might be constrained by pre-existing factors. To correct for multiple comparisons where appropriate, the false discovery rate (FDR) (57) was used. Reported *p*-values already correspond to the correction. All major statistical analyses were conducted using SPSS 25 (IBM Corp. Released 2017. IBM SPSS Statistics for Windows, Version 25.0. Armonk, NY: IBM Corp.). The statistical significance level was set to α = 0.05.

## Results

### Sample Characteristics

Detailed sociodemographic characteristics are found in Table 1. Overall, a total of 86 children and adolescents between the ages of 11 and 19 with SITB participated in time point 2. An additional number (*n* = 7) were recruited but could not be included in the sample as they either showed none of the critical variables (*n* = 1), failed to come to the time points (*n* = 2), had language difficulties (*n* = 3), or eventually decided not to take part in the study (*n* = 1). The presented data were collected from July 2019 to November 2020. As mentioned above, the mean age was *M* = 15;8 (years; months) (*SD* = 1;8, range = 11;3–18;3) and 75.6% were female. Girls and boys did not differ in age (*t*_(86)_ = 0.12, *p* = 0.322).

**Table 1:** Sociodemographic characteristics of participants at time point 2.

			**Total *N***
**Sex**	***N***	**%**	86
Female	65	75.6	
Male	21	24.4	
**Age**	***M***	***SD***	86
	15;8	1;8	
**School type**	***N***	**%**	86
Gymnasium	16	18.6	
Realschule	24	27.9	
Mittelschule	19	22.1	
Förderschule	3	3.5	
Berufsschule	10	11.6	
Other/No school	7	8.1	
Unknown	7	8.1	
**Parental relationship status**	***N***	**%**	76
Live together	32	37.2	
Separated/divorce	36	41.9	
Separated by death	1	1.2	
Never lived together	7	8.1	
**Household composition**	***N***	**%**	
With biological mother	58	87.8	66
With other mother figure	3	4.5	
With no mother/mother figure	5	7.7	
With biological father	37	63.8	58
With other father figure	13	22.4	
With no father/father figure	8	13.8	
With mother/father	68	93.1	73
At institutional care	4	5.5	
Lives with partner	1	1.4	

*Gymnasium (higher level education, usually 8–9 years of school after 4 years of elementary school, terminating with the general university entrance qualification), Realschule (intermediate secondary school, 6 years of school after 4 years of elementary school), Mittelschule (9 years of elementary school), Berufsschule (2 to 3 years vocational training school most commonly after Mittelschule or Realschule, but also possible after Gymnasium) and Förderschule (special needs school)*.

The distribution of psychiatric diagnoses according to ICD-10 (sorted by its frequency) is as follows: F3 (Mood [affective] disorders), *n* = 74; F4 (Neurotic, stress-related and somatoform disorders), *n* = 49; F9 (Behavioral and emotional disorders with onset usually occurring in childhood and adolescence), *n* = 26; F1 (Psychological and behavioral disorders caused by psychotropic substances), *n* = 12; F6 (Disorders of adult personality and behavior), *n* = 5; F5 (Behavioral syndromes associated with physiological disturbances and physical factors), *n* = 3 and F8 (Disorders of psychological development), *n* = 3. It should be taken into account that several diagnoses were possible per patient (mean number of diagnoses: 2.3).

The SCID was used to determine the presence or absence of a borderline personality disorder among the investigated group of patients and *n* = 2 patients qualified for a borderline personality disorder.

Overall, details on clinical variables including the dependent variables can be found in Table 2.

**Table 2:** Clinical characteristics of participants at time point 2.

				**Total *N***
**SCID (Borderline-Personality Disorder section)**	***N***	**%**	**Range**	80
3 fulfilled criteria	12	15.0		
4 fulfilled criteria	15	18.8		
>5 fulfilled criteria	2	2.6		
**SCL-90-R: GSI**	***M***	***SD***		82
	1.23	0.66	0.02–3.03	
**GAF**	***M***	***SD***		86
	48.0	8.13	35–72	
**CGI-S**	***M***	***SD***		86
	3.64	0.57	3–5	
**ERS**	***M***	***SD***		84
Sensitivity	21.33	9.73	0–3.9	
Arousal/Intensity	14.55	7.10	0–3.86	
Persistence	7.75	3.92	0–4	
Total	43.64	19.59	0.43–11.01	
**Types of NSSI reinforcement**	***M***	***SD***		68
Automatic positive reinforcement	3.04	1.07	0–4	
Automatic negative reinforcement	2.29	1.55	0–4	
Social positive reinforcement	0.35	0.84	0–4	
Social negative reinforcement	1.22	1.35	0–4	
**Suicidal thoughts**	***N***	**%**		85
Prevalence	79	92.9		
	***M***	***SD***		79
Lifetime (number of episodes)	13.39	21.01	0–100	
Last year (number of episodes)	6.0	8.50	0–40	
**Suicide attempts**	***N***	**%**		83
Prevalence	27	32.5		
	***M***	***SD***		82
Lifetime (number of episodes)	0.65	1.22	0–5	
Last year (number of episodes)	0.43	0.92	0–3	
**NSSI thoughts**	***N***	**%**		84
Prevalence	72	85.7		
	***M***	***SD***		
Lifetime (number of episodes)	44.26	105.40	0–500	76
Last year (number of episodes)	30.47	76.53	0–398	79
**NSSI behavior**	***N***	**%**		84
Prevalence	69	82.1		
	***M***	***SD***		
Lifetime (number of episodes)	38.03	88.71	0–600	73
Last year (number of episodes)	19.86	34.35	0–200	73

*The ERS (Emotion Reactivity Scale) scores reported are the projected ERS scores for compatibility. Mean scores were used for the analysis. The four reported types of NSSI reinforcement are items from the Self-Injurious Thoughts and Behaviors Interview (SITBI) with a maximum score of 4 which represents agreement. SCID, Structured Clinical Interview for DSM-IV; Axis II; SCL-90-R, Symptom Checklist-90 Revised; GSI, Global Severity Index; GAF, Global Assessment of Functioning Scale; CGI-S, Clinical Global Impressions Scale*.

### Family History and Peers' History of NSSI

In order to determine the effects of sex, positive family history, and positive peers' history, Mann-Whitney-*U*-tests were computed for the four dependent variables (suicidal ideation, suicide attempts, NSSI thoughts, NSSI behavior). This was done for the patient's lifetime prevalence since sex and family history are factors to be present early on. There was no effect of sex or positive peers' history, however, there was a significant effect of family history on suicidal ideation, indicating that suicidal ideation was significantly greater for positive family history (*Mdn* = 10) than for a negative family history (*Mdn* = 2). See Table 3 for the detailed *U* and *p*-values for the whole set of variables. The effect of age was determined through bivariate correlations between age calculated in days at time point 2 and the suicidal ideation/attempts and NSSI thoughts/behavior. As patients' age ranged from 11 to 18 and thus older patients had more time to experience suicidal ideation and NSSI, the outcome variables for the past year were chosen. There was no significant correlation between age and any of the outcome variables (τ = −0.12, −0.01, *p* > 0.1).

**Table 3:** Results of Mann-Whitney-*U*-tests for dependent variables through lifetime.

**Dependent variables**	**Group variable**	***N***	**Mean rank**	***U***	***P*-value**
Suicidal ideation	Sex	79	Male = 41.42 Female = 39.55	543.00	0.756
	Family history	69	Neg = 28.26 Pos = 41.18	816.50	0.021
	Peers' history	77	Neg = 40.63 Pos = 37.71	675.50	0.756
Suicidal attempts	Sex	82	Male = 34.80 Female = 43.66	754.00	0.224
	Family history	72	Neg = 36.36 Pos = 36.64	653.00	0.942
	Peers' history	80	Neg = 37.68 Pos = 42.81	893.500	0.337
NSSI thoughts	Sex	76	Male = 34.80 Female = 39.82	634.00	0.381
	Family history	67	Neg = 27.82 Pos = 39.01	740.50	0.057
	Peers' history	75	Neg = 33.23 Pos = 41.75	850.50	0.381
NSSI behavior	Sex	73	Male = 30.02 Female = 39.63	669.50	0.249
	Family history	65	Neg = 31.12 Pos = 34.82	588.00	0.429
	Peers' history	72	Neg = 32.66 Pos = 39.58	763.00	0.241

*p-values have been corrected according to the false discovery rate (FDR). Positive (Pos) Family History, suffering from neurological diseases or mental illness and specifically attempted suicide/completed suicide and NSSI in family members. Negative (Neg) Family History, no suffering from neurological diseases or mental illness and specifically attempted suicide/completed suicide and NSSI in family members. Positive (Pos) Peers' History, occurrence of suicide attempts or NSSI among close friends. Negative (Neg) Peers' History, no occurrence of suicide attempts or NSSI among close friends*.

**Figure 1:**
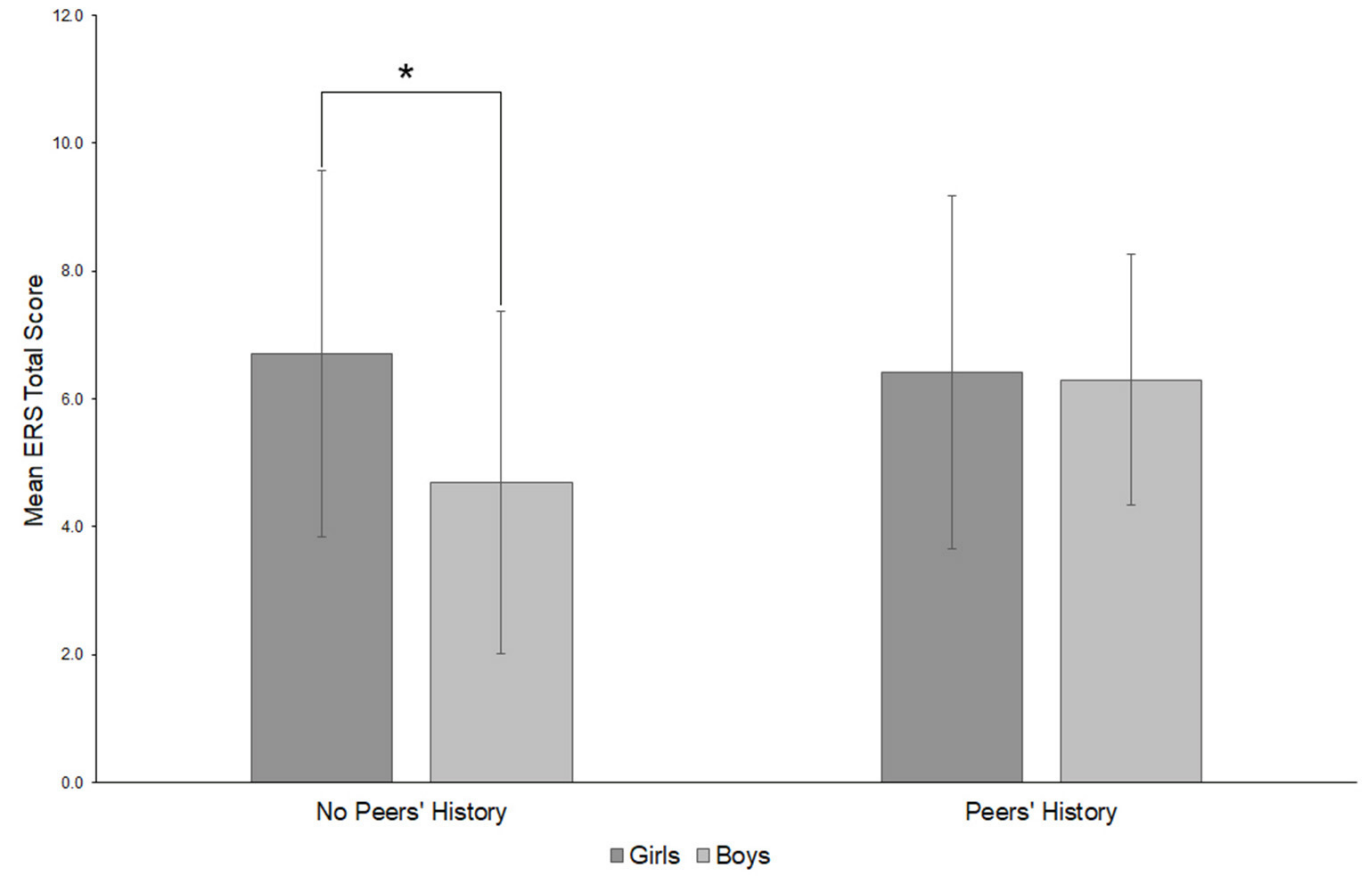
*Post-hoc* overview of the interaction between sex and having close friends with a history of suicide attempts or NSSI (peers' history) (on ERS mean scores). *p < 0.05. Emotional Reactivity Scale (ERS).

The effect of sex, family and peers' history and the covariate age on the ERS score was examined separately through a Three-Way ANOVA. The main effect of the covariate age on the ERS score was statistically significant, *F*_(1, 64)_ = 10.95, *p* = 0.002, η^2^ = 0.146 (large), but no other main effects. There was a significant interaction effect between sex and peers' history, *F*_(1, 64)_ = 4.74, *p* = 0.033, η^2^ = 0.07 (medium). *Post-hoc* tests revealed a significant difference between boys and girls when no peers' history is present (*t*_(34)_ = 2.07, *p* = 0.049) with boys having lower ERS scores (*M* = 4.69, *SD* = 2.68) than girls (*M* = 6.71, *SD* = 2.86), but no difference when both groups had positive peers' history [*t*_(44)_ = 0.12, *p* = 0.907]. See Figure 1 for a graphical overview of the interaction effect.

### Emotional Reactivity and NSSI

As there was a significant positive correlation between age and the ERS score (τ = 0.25, *p* = 0.019), the relationship between ERS and the outcome variables was examined through partial correlations while controlling for age. As above, only the relationship for outcome variables over the period of 1 year were examined since older patients had more opportunities to experience suicidal ideation and NSSI throughout lifetime. There were no significant correlations between ERS scores and suicidal ideation, suicide attempts, and NSSI thoughts. However, there was a significant correlation between the ERS scores and NSSI behavior within the past year (see Table 4 for an overview of correlations). Only the correlation with the ERS sensitivity score remained significant in a partial correlation when controlling for age (*r* = 0.28, *p* = 0.029). Thus, the ERS sensitivity score was considered relevant for the prediction of NSSI behavior. A simple linear regression was calculated to predict NSSI behavior as dependent variable in the past year based on the ERS sensitivity score as independent variable. A significant regression equation was found [*F*_(1, 70)_ = 4.77, *p* = 0.032)] with an *R*^2^ of 0.06. Number of NSSI behaviors increased 8.95 for each score point of ERS sensitivity. Since the relationship remained significant when controlling for age in a partial correlation, age was not added as an independent variable into the regression. However, also when adding age as an independent variable, only ERS sensitivity remained as a significant predictor.

**Table 4:** Correlation matrix for study variables.

**Variable**	**1**	**2**	**3**	**4**	**5**	**6**	**7**	**8**	**9**	**10**	**11**	**12**
1. Suicidal thoughts past year	–											
2. Suicide attempts past year	0.24^*^	–										
3. NSSI thoughts past year	0.18^*^	0.05	–									
4. NSSI behavior past year	0.14	0.10	0.38^*^	–								
5. ERS total score	0.01	−0.12	0.14	0.25^**^	–							
6. ERS sensitivity	0.03	−0.14	0.16^*^	0.25^**^	0.82^**^	–						
7. ERS arousal/intensity	−0.01	−0.13	0.14	0.22^*^	0.81^**^	0.74^**^	–					
8. ERS persistence	−0.00	−0.09	0.10	0.24^**^	0.76^**^	0.63^**^	0.59^**^	–				
9. Automatic negative reinforcement	−0.06	−0.08	−0.06	0.12	0.15	0.15	0.08	0.19	–			
10. Automatic positive reinforcement	0.02	0.85	−0.05	0.22^*^	0.22^*^	0.19^*^	0.17	0.25^*^	0.39^***^	–		
11. Social positive reinforcement	0.09	−0.14	0.17	−0.03	0.19	0.18	0.20	0.18	−0.05	0.06	–	
12. Social negative reinforcement	0.07	0.09	0.04	0.23^*^	0.27^*^	0.27^*^	0.30^*^	0.20	0.05	0.22^*^	0.14	–

*Correlation coefficients correspond to Kendall's τ. An FDR correction has been applied*.

* *p < 0.05*,

** *p < 0.01*,

*** *p < 0.001*.

To examine the relationship between ER and the outcome variable NSSI behavior in the past year in more detail, additional correlations with automatic positive reinforcement, automatic negative reinforcement, social positive reinforcement, and social negative reinforcement were computed. After correcting for multiple comparisons, only automatic positive reinforcement (τ = 0.22, *p* = 0.034) and social negative reinforcement (τ = 0.27, *p* = 0.016) significantly correlated with the ERS score. Only the correlation with social negative reinforcement remained significant when controlling for age in a partial correlation (*r* = 0.35, *p* = 0.005). When considering the subscales, automatic positive reinforcement was only related to the ERS sensitivity score and the ERS persistence score, whereas social negative reinforcement was related to the ERS sensitivity scale and the ERS arousal/intensity scale (see Table 4). When controlling for age in partial correlations, the correlation between the automatic positive reinforcement and the ERS sensitivity score (*r* = 0.65, *p* = 0.042) and the ERS persistence score (*r* = 0.35, *p* = 0.004) remained significant and increased in strength. For social negative reinforcement partial correlations revealed significant correlations with both ERS subscales when controlling for age (ERS sensitivity: *r* = 0.33, *p* = 0.008; ERS arousal/intensity: *r* = 0.34, *p* = 0.005). All partial correlations increased in effect size upon controlling for age. Interestingly, even though all four items specifically address reasons for NSSI, only the two items that were correlated with the ERS were also significantly correlated with the outcome variable NSSI in the past year.

### Reasons Triggering the Crisis

In order to determine the degree of agreement between guardians and patients on reasons for the emergency presentation (guardians) and for NSSI behavior (patients), Cohen's κ was computed. Agreement was determined for the surveyed reasons. See Table 5 for level of agreement for the different reasons. Overall, agreement was significant on about 60% of the reasons. However, significance was tested against 0, thus, mean agreement was only at 0.38, which constitutes only fair agreement (58). Reasons on which no significant agreement was present were problems with friends, problems with physical health and problems with mental health. For these reasons also κ_max_ was reduced which indicates a portion of agreement that cannot be achieved due to pre-existing factors which produce unequal marginal totals.

**Table 5:** Cohen's κ and proportion of agreement between guardians and patients on reasons for emergency presentations in respect to NSSI behavior.

**Reasons**	**Cohen's κ**	***P*-value**	**κ_max_**
School	0.33	0.009	0.97
Family	0.34	0.005	0.72
Friends	0.18	0.140	0.75
Romantic partner	0.30	0.015	0.88
Finances	0.89^a^		0.98
Bullying	0.25	0.040	0.80
Physical health	0.06	0.609	0.74
Death of a person	0.68	<0.001	0.68
Mental health	0.03	0.790	0.71
Humiliating experience	0.39	0.002	0.93

*Correlation coefficients correspond to Kendall's τ. An FDR correction has been applied*.

a *observed concordance was smaller than mean-chance concordance, therefore proportion of agreement was calculated instead of κ*.

## Discussion

The major purpose of the present study was to report a cross-sectional analysis of the phenomenology and family-related factors of adolescents with SITB presenting to a specialized emergency out-patient setting within a clinic for child and adolescent psychiatry.

Whereas, in our sample 75.6% of the patients were female and the mean age was 15;8 (years; months), Porter et al. (5) showed no observable sex difference (51% male) with a mean age of 14.5. We included the emergency clientele from the age of 11 onward, which explains the higher average age. The above-mentioned study (5) deals with patients under 18 years who visited the emergency pediatric department who needed psychiatric evaluation, while in our case a distinct selection was made of NSSI and suicidal behavior in a child and adolescent psychiatric setting. The higher proportion of girls has also been confirmed in other studies on NSSI and suicide attempts in adolescents (20, 59, 60).

We also interviewed the custodians of our adolescent patients about the psychiatric history of family members. We identified a significant effect of family history on suicidal ideation, indicating that suicidal ideation was significantly greater for positive family history. Regarding suicidal ideation and acts of suicide, previous research (26) has shown corresponding results. It has been found that the rate of attempted suicide was higher among first-degree relatives of adolescent suicide participants compared to the relatives of controls (61). First-degree relatives of suicide completers had twice as much suicidal ideation as the relatives of control persons (61). It has been shown that there is an association between familial transmission of suicidal ideation and transmission of psychiatric disorders. But the essential liability to respond to suicidal ideation was discussed as a significant mechanism for suicidal behavior transmitted within the family (26). The reason that our sample only shows an effect on suicidal ideation could be due to recent and newly emerging symptoms. As Glenn et al. (23) showed that the earliest age of entry was at NSSI and suicidal ideation, followed by NSSI behavior, suicide plans and attempted suicides (23), it is possible that the transition from suicidal ideation to suicidal behavior could not yet have reached its full extent in our sample. In addition, our sample has been selected in such a way that we have not included any patients who are especially burdened and require a longer inpatient stay due to their symptoms, nor have we included any patients who already had adequate child and adolescent psychiatric care.

*Post hoc* tests revealed a significant difference between boys and girls when no peers' history is given with boys having lower ERS scores than girls, but no difference when both groups had a positive peers' history (i.e., the teenagers report that close friends show NSSI and/or attempted suicide). Adolescents with a history of NSSI most often named their peers as source for the idea of hurting themselves (62). Our results may indicate that for boys close friends who did not report suicide attempts and/or NSSI function as a protective factor and consequently they show lower ER. For girls, this assumed protective factor could not be found in our sample. Consequently, it would be important and useful to focus on a possible sex difference in further research.

We identified a significant correlation between the ERS sensitivity score and NSSI behavior within the last year, independent of age. This indicates that responding to many stimuli is what is demanding, not the intensity or duration of the emotions. According to Nock et al. (38), ER refers to the extent to which an individual experiences emotions. Our results show that an emotional reaction to a wide range of stimuli (e.g., emotion sensitivity) is the only explanatory factor among the ERS scale for NSSI behavior during the last year. Previous research showed that young adults who engage in NSSI showed significantly higher emotional sensitivity, arousal/intensity, and persistence than a control group (39). Overall, ER appears to be only one variable that affects NSSI in this present study (40). Numerous anamnestic, but also psychopathological variables can influence it (38). Nock et al. (38) showed significantly higher ER in adolescents with a mood, anxiety, or eating disorders compared to people without such disorders. There are latent subgroups of individuals who engage in NSSI (adults) with different difficulties in emotion regulation (63). It is questionable whether this differentiation is already present in adolescent patients, or whether the psychopathology only develops in this way throughout puberty. This statement is supported by the fact that our results show that the older the patients are, the higher the ERS scores. According to analyses by Lannoy et al. (64) there was no significant relationship between ER and age; there, however, an adult sample (participants from the community) were examined. In addition, the results of a study that investigated the course of emotion regulation strategies in different age ranges indicate a general trend toward increasing adaptive emotion regulation (65). Specifically, middle-aged adolescents showed the smallest repertoire of emotion regulation strategies. In our study, the average age is also located in middle adolescence. The above-mentioned study sample included healthy subjects from young adolescent to middle-aged adults. It could be assumed the described temporal sequence occurs with a delay in people with NSSI and/or suicidal behavior.

A relationship between the ERS and distinct types of reinforcement (automatic positive reinforcement, social negative reinforcement) as a motivation factor for NSSI was found. This suggests that for automatic positive reinforcement it is especially the sensitivity to various kinds of stimuli and the persistence of these feelings that plays a crucial role, whereas for social negative reinforcement it is the sensitivity to various kinds of stimuli and the intensity of these feelings. The most strongly affirmed function for each form of SITB was according to the results of Nock et al. (49) automatic negative reinforcement, that was followed by automatic positive reinforcement, emphasizing the relevance of these functions and suggesting that different forms of SITB may serve somewhat similar functions (49). The only aberration from this pattern was with regard to suicidal gestures, which adolescents reported using for social reinforcement (49). Up to this point, research has been less focused on examining the intrapersonal positive or interpersonal negative reinforcement functions of self-injury, and these continue to be pivotal directions for future research (7). The two intrapersonal functions can be particularly relevant in the case of individuals with emotional dysregulation and it was shown that the two intrapersonal functions can be merged into an affect regulation function. In addition, we found the correlation between ER, negative social reinforcement and NSSI of special interest. In our experience, this aspect also plays a subordinate role in everyday clinical practice and should be investigated more closely in the context of future therapy research on NSSI.

We were able to confirm the hypothesis that the reasons given by caregivers for the current crisis might be different from the reasons given by patients for NSSI behavior. Especially if the trigger for the crisis is for example a dispute with the family/parents, differences in agreement are of great relevance for further therapy planning. Therefore, the parents' point of view should not be overestimated, but also the patients' opinion should be investigated. Otherwise, it could happen that the influence of the family situation, but possibly also other factors (i.e., problems with friends, problems with physical health, and problems with mental health) which might be responsible for triggering the crisis, are underestimated. The results of Fu et al. (15) demonstrated that parents lack knowledge about NSSI and its treatment and suffer from great emotional stress (66). In addition to this lack of knowledge, an aggravating factor is that according to our results, parents partly assume other causes for the crisis compared to their children. This could either result from the fact that patients are more reserved toward their caregivers and parents cannot know any better. Or perhaps the caregivers tend to externalize reasons for the crisis. Especially the reasons problems with friends, problems with physical health and problems with mental health are topics that can probably also be better judged by the patients themselves than the guardians, since these are topics that concern the young people themselves and are rather difficult to assess by the caregivers. When parents and their children appraise the child's emotional and behavioral health problems, their valuations are often divergent (67), especially for internalizing problems (68). For example, relying only on parents to identify depression in children may lead to young people with depression being overlooked and therefore going undetected as well as untreated (69, 70). Our findings show that it is particularly important to consider family dynamics and other contextual factors when choosing the appropriate therapy for youths with self-injurious behavior (71).

Although our study addresses a patient clientele that increasingly requires emergency care in child and adolescent psychiatric clinics and outpatient services, there are some limitations regarding this study. The psychiatric assessments were performed by four experienced and trained clinicians and final decision on categorical diagnoses has been made including certified child psychiatrists. A limitation to be mentioned here is that no interrater reliability was calculated. However, these colleagues were carefully instructed and trained in conducting the interviews. An additional aspect that can be considered a limitation is that the number of SITB episodes shows a large range. This is due to patients providing numerical values to the questions. Measures that rely on patients' self-report may suffer from a bias and especially in clinical contexts there are not many objective ways to acquire frequency estimates of NSSI behavior. Still, in cases with unrealistic replies clinicians ensured to acquire the most realistic response possible. For a comprehensive standardized diagnostic procedure within the context of an acute psychiatric setting, the number of subjects has reached a respectable size. However, the current sample was drawn from a single psychiatric clinic and regional differences across countries may be possible. Future studies with a similar sample might be able to extend the current findings to international contexts. Finally, in our sample, patients with long-term inpatient child and adolescent psychiatric treatment needs and patients who already had adequate outpatient care were excluded. Therefore, our results do not reflect a complete utilization sample of an emergency department for SITB. This in turn has an impact on the results since including these subgroups might have led to more abnormalities/pathologies in our sample of emergency outpatient patients. Still, the current sample can be considered representative for outpatients of a psychiatric clinic for children and adolescents that do not require long-term inpatient care but nevertheless have a strong need for interventions.

However, a major strength of our study is that our examination of the outpatients in the emergency department and evaluation of their reasons/motivation for SITB took place without delay. In addition, we have used a clinical gold standard for specified psychiatric assessments, especially with the structured clinical interviews M.I.N.I. KID and SITBI, conducted by experienced clinicians. In addition to symptoms regarding NSSI and suicidal ideation and behavior, we have also addressed questions which are relevant for patient care and future treatments. We identified ER as a relevant aspect of NSSI and extended these findings by relating them to distinct types of reinforcement in respect to NSSI. Especially social negative reinforcement has rarely been emphasized and its relation to ER has not been examined previously. Furthermore, we investigated the influence of positive family and peers' history on self-injurious behavior of patients and compared patients' statements with those of their caregivers regarding the causes of the crisis. The results of the present study may facilitate future research on risk and influencing factors in adolescents with SITB as determined in this sample.

## Data Availability Statement

The raw data supporting the conclusions of this article will be made available by the authors, without undue reservation.

## Ethics Statement

The studies involving human participants were reviewed and approved by ethic commission of the University of Regensburg. Written informed consent to participate in this study was provided by the patients and the participants' legal guardian/next of kin.

## Author Contributions

SK and RB had the idea for the study and developed the study design. IJ contributed to hypotheses, sample size calculation, and statistical analyses. First manuscript has been written by SK and IJ. AE, DS, and AO participated in the design and coordination of the study. As a clinical collaborator, JM plays a major role in patient coordination and has conducted a significant proportion of time points with patients. All authors read and approved the final manuscript.

## Conflict of Interest

The authors declare that the research was conducted in the absence of any commercial or financial relationships that could be construed as a potential conflict of interest.
